# Isolation, characterization and hypolipidemic activity of ferulic acid in high-fat-diet-induced hyperlipidemia in laboratory rats

**DOI:** 10.17179/excli2016-394

**Published:** 2016-10-25

**Authors:** Pankaj G. Jain, Sanjay J. Surana

**Affiliations:** 1 R. C. Patel Institute of Pharmaceutical Education and Research, Shirpur, Dist-Dhule-425405, Maharashtra, India

**Keywords:** Prosopis cineraria, column chromatography, oxidative stress, nitric oxide, lipid profile

## Abstract

*Prosopis cineraria *(L.) Druce (Leguminosae) (syn. *Prosopis spicigera *L.) has antidiabetic and antioxidant potential. Earlier we reported its hypolipidemic activity obtained from ethanol extract (ET-PCF). Object of this work was to isolate ferulic acid (FA) from ET-PCF and evaluate hypolipidemic activity against high-fat diet (HFD)-induced hyperlipidemic laboratory rats. ET-PCF was subjected to flash column chromatography to isolate FA. The chemical structure of the isolated compound was elucidated by UV, IR, ^1^H NMR,^13^C NMR and LC-MS. Further, the antihyperlipidemic effect of FA (10, 20 and 40 mg/kg, p.o.) in HFD-induced hyperlipidemic rats was investigated. Hyperlipidemia was induced in male Sprague-Dawley rats by feeding with HFD for 60 days. Lipid parameters such as total cholesterol (TC), Low-density lipoprotein cholesterol (LDL-C), high-density lipoprotein cholesterol (HDL-C) and triglycerides (TG) levels were measured in serum and hepatic tissue. Hepatic oxido-nitrosative stress (SOD, GSH, MDA and NO) were also determined. Histological evaluation of liver tissue was carried out. The structure of the isolated compound was characterized based on spectral data and confirmed as FA. HFD induced an alteration in serum, and hepatic lipid profile (triglyceride, cholesterol, HDL, and LDL) was significantly restored (p *< *0.001) by administration of FA (20 and 40 mg/kg, p.o.). The elevated level of oxido-nitrosative stress in liver was significantly reduced (p *< *0.001) by FA (20 and 40 mg/kg, p.o.). Histological aberration induced in the liver after HFD ingestion were restored by FA administration. Ferulic acid isolated from ET-PCF showed hypolipidemic effects in HFD-induced hyperlipidemic rats via modulation of elevated oxido-nitrosative stress.

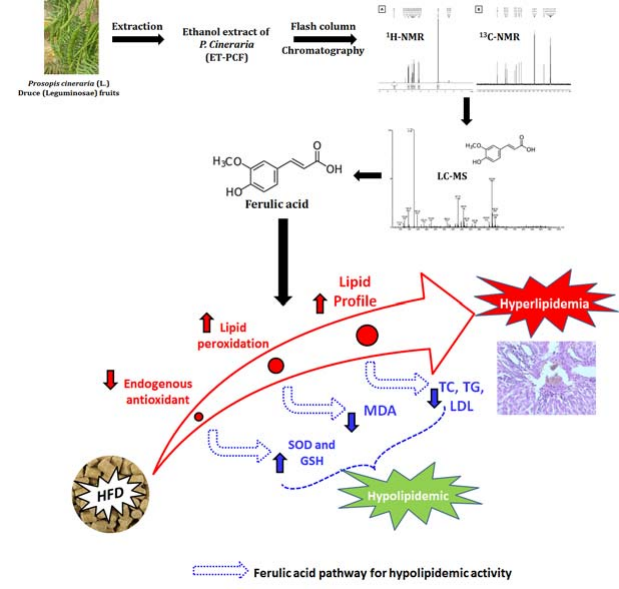

## Introduction

Maladies of the cardiovascular system are a prominent health hazard often leading to death. Sedentary lifestyle and erroneous food habits encompassing consumption of fats and refined sugars lead to a decrease in the activity of myosin and actin filaments (Brai et al., 2007[7]) accentuate the risk of hyperlipidemia, a noteworthy risk factor for well-known cardiovascular disorders, atherosclerosis, and allied cardiovascular complexities. Around 17.5 million people have died due to cardiovascular disease (CVD) representing 31 % of all global deaths and it is expected to grow to more than 23.6 million by 2030 (Mozaffarian et al., 2016[37]). The proportion of hypertensive individuals in India is anticipated to expand 2-fold by 2025 (Brai et al., 2007[7]). The ischemic coronary disease is a noteworthy risk factor in the pathogenesis of preoperative adverse cardiovascular events which enhance the risk of mortality in a high-risk surgical patient sub-population (Howard-Alpe et al., 2006[15]).

Patient's cholesterol profile plays a decisive role in the treatment of hyperlipidemia. Numerous antihyperlipidemic moieties including statins, fibrates, niacin, bile acids, ezitimibe, etc. decrease cholesterol levels albeit by a different mechanism (Durrington, 2003[12]). At present, available hypolipidemic medications provide relief in a fraction of patients and have been associated with an array of side effects including hyperuricemia, diarrhea, nausea, myositis, gastric aggravation, flushing, dry skin and altered liver function (Santharam et al., 2015[43]).

For the treatment of various diseases such as metabolic syndrome, diabetes, and cancer, various plant containing polyphenols have been widely used as a potential source of treatment (Williamson and Manach, 2005[51]). Ferulic acid (4-hydroxy-3-metoxybenzene acrylic acid), a natural polyphenolic compound, is derived from a wide variety of plants such as tomato, citrus fruits, carrot, sweet corn, cabbage, broccoli, bananas and rice bran (Zhao and Moghadasian, 2008[53]). Moreover, cell wall of cereal grains and a variety of food plants (pineapple, bananas, spinach, and beetroot) contain extractable amount (0.5-2 %) of FA, and flax seed contains the highest concentration of ferulic acid glucoside (4.1 ± 0.2 g/kg) (Besseau et al., 2007[5]; Kumar and Pruthi, 2014[32]). FA is relatively easily and well absorbed when compared to other flavonoid compounds (Bourne et al., 2000[6]). It has been reported that ferulic acid (FA) has a vital cytoprotective role and proficiently neutralize the onset and propagation of a wide cluster of diseases, especially of neurodegenerative organs (Mancuso et al., 2007[34]). FA has been reported to have anticancer, antihyperlipidemic, antihypertensive, antimicrobial, antiviral, radioprotective, cardioprotective, antidiabetic, anti-inflammatory, antiulcer and hepatoprotective potential (Baskaran et al., 2010[4]; Choi et al., 2011[9]; Liu et al., 2012[33]; Marimuthu et al., 2013[35]; Prasad et al., 2006[41]). It has been reported that FA acts as a β-secretase modulator with therapeutic potential against Alzheimer's disease (Mori et al., 2013[36]). It is a potent scavenger of hydroxyl and peroxyl radicals in brain cells and macrophages, a property that could be responsible for its defensive capacity (Kim et al., 2004[31]; Ogiwara et al., 2002[39]). FA promptly frames a resonance-stabilized phenoxy radical, which represents its potent antioxidant activity (Kikuzaki et al., 2002[29]; Ogiwara et al., 2002[39]).

Of late, a few experimental animal models of hyperlipidemia have been developed to characterize distinctive segments of the pathophysiological forms that portray this disease. In the present study, hyperlipidemia in rats was induced by administration of HFD over a period of 60 days. It has been proven that HFD caused a significant elevation in HDL-C in clinical as well as preclinical studies (Katan et al., 1994[27]; Nevin and Rajamohan, 2009[38]). The previous study carried out in our laboratory found that the ethanol extract of *Prosopis cineraria *(L.) Druce (*Leguminosae*) (syn. *Prosopis spicigera *L.) possesses potent anti-hyperlipidemic action against high-fat-diet-induced hyperlipidemia in laboratory rat (Jain and Surana, 2016[17]). However, purification, standardization and anti-hyperlipidemic activity evaluation of ethanol extract of *P. cineraria* (ET-PCF) is not yet reported. Therefore, the present study was undertaken with an objective of isolation and characterization of ET-PCF and its evaluation for antihyperlipidemic effect on experimental laboratory rats. 

## Materials and Methods

### Drugs and chemicals

Various drugs and chemicals were obtained from a commercially available manufacturer such as HFD (60 kcal % fat, # D12492, 5.24 kcal/g, Research Diet Inc., New Brunswick, NJ, USA). Cholesterol, triglyceride, HDL-C and LDL-C kits (Accurex Biomedical Pvt. Ltd., Mumbai, India), Petroleum ether (60:80) and diethyl ether (Merck, India). 

### Collection of plant material

The fresh fruits of the plant *P. cineraria *were collected from Satpuda region of Maharashtra, India. Professor L. K. Kshirsagar (Taxonomist, Department of botany, S.S.V.P.S's L. K. Dr. Ghogre Science College, Dhule, North Maharashtra University, Jalgaon) was authenticating the plant. 

A specimen of the same has been submitted to the herbarium of the division.

### Preparation of ethanol extract and isolation

Preparation of ethanol extract of *P. cineraria* fruit was carried out according to the previously reported method (Jain and Surana, 2015[18]). Briefly, cleaned fruits of *P. cineraria *were cut into small pieces and shade dried. Then it crushed in a grinder and pulverized into fine powder. This powdered material (500 g) was passed through 40 meshes and macerated with 70 % ethanol using Soxhlet extractor. The ethanol extract of *P. cineraria* fruits (ET-PCF) was concentrated in vacuum evaporator below 40 °C. The yield obtained was 12.2 % w/w, and it was used for isolation of FA. 50 gram of ET-PCF was poured on the top of the column packed with silica gel. Elution mixture of isoamyl alcohol:acetic acid:water (1:1:2) to afford a light yellow color solid compound (1.34 g). It was recrystallized from hexane to yield 0.68 g of product. Thin layer chromatography of ET-PCF in isoamyl alcohol:acetic acid:water (1:1:2) showed the presence of yellowish green colored single spot (Rf 0.88). The remaining fruit powder was soaked in ethanol for another six days. The solution was filtered, and the combined filtrates were concentrated using a rotary evaporator to yield 60.3 g of a brown oily bark ethanol extract. This ET-PCF underwent silica gel flash column chromatography (Merck 1.07747) using 10 % polarity increments from 90:10, chloroform:ethanol to 100 % ethanol whereby 100 mL fractions were collected. The flash column chromatography fractions 5, 6 and 7 were combined (2.7 g) and subjected to radial chromatography to produce five fractions. The combined fractions 1, 2 and 3 were fractionated over silica gel (Merck 1.07749) on preparative thin layer chromatography to give 54 mg of product.

### Chemical characterization of isolated molecule

The technique such as UV, IR, ^1^H NMR,^ 13^C NMR and LC-MS were used to determine the chemical structure of the isolated compound. IR spectrum was recorded using KBr pellets on a Perkin-Elmer IR spectrometer (Perkin-Elmer, Waltham, MA). ^1^H NMR and ^13^C NMR spectra were recorded using CDCl_3_ as solvent on Bruker Advance II 400 NMR and LC‐MS spectra were recorded at high resolution on a mass spectrometer (Perkin Elmer Auto system) at spectrometer SAIF Panjab University, Chandigarh, the data are given in m/z values.

### Experimental animals

Male Sprague-Dawley rats (180-220 g) were obtained from college animal house of R. C. Patel Institute of Pharmaceutical Education and Research, Shirpur, India. They were housed in well-ventilated cages and maintained at a controlled temperature of 22 ± 2 °C with a 12 h light/dark cycle and standard lab control. The animals had free access to standard pellet chow (Pranav Agro-Industries Ltd., Sangli, India) and filtered water *ad libitum* throughout the experimental protocol. Institutional Animal Ethical Committee of RCPIPER College, Shirpur approved the study protocol (IAEC/RCPIPER/ 2012-13/09).

### Development of high-fat diet fed rats

The two dietary regimes such as normal pellet diet (NPD) and high-fat diet (HFD, 58 % fat, 25 % protein, and 17 % carbohydrate, as a percentage of total kcal, *ad libitum*) were fed to rats for the initial period of 60 days. The composition and preparation of HFD as were described elsewhere (Jain and Surana, 2016[17]). 

### Experimental design

The studies were conducted in the following groups of animals

- Group I: Normal rats: rats received normal pellet diet, and they were treated with vehicle (10 mg/kg of distilled water)
- Group II: HFD control: rats received high-fat diet, and they were treated with vehicle (10 mg/kg of distilled water)
- Group III: AT (1.2): rats received high-fat diet, and they were treated with atorvastatin (1.2 mg/kg)
- Group IV: FA (10): rats received high-fat diet, and they were treated with ferulic acid (10 mg/kg)
- Group V: FA (20): rats received high-fat diet, and they were treated with ferulic acid (20 mg/kg)
- Group VI: FA (40): rats received high-fat diet, and they were treated with ferulic acid (40 mg/kg).

The dose of FA (10, 20 and 40 mg/kg, p.o.) was selected on the basis of a previously reported method (Balasubashini et al., 2004[3]). Vehicle or atorvastatin or FA was administered orally for 60 consecutive days. After the end of treatment, rats fasted overnight and, after 24 h, they were sequentially anesthetized with anesthetic ether for about 30-40 s. The blood was withdrawn by retro-orbital puncture. Each blood sample was collected into separate vials for the determination of serum parameters.

### Preparation of serum samples and biochemical estimations

The serum was separated by centrifugation using an Eppendorf cryocentrifuge (model no. 5810, Eppendorf, Hamburg, Germany), maintained at 4 °C and run at a speed of 7000 rpm for 15 min. The levels of high-density lipoprotein (HDL), low-density lipoprotein (LDL), triglyceride (TG) and Total cholesterol (TC) were measured by a spectrophotometer (UV-visible spectrophotometer, Jasco V-530, Tokyo, Japan) using commercially available reagent kits according to procedure provided by manufacturer (Accurex Biomedical Pvt. Ltd., Mumbai, India) (Adil et al., 2015[1]; Honmore et al., 2015[14]).

### Preparation of liver homogenates

After the blood collection, the animal was sacrificed by cervical dislocation and the liver was removed and immediately homogenized in 3 mL of ice-cold PBS buffer. One portion of the supernatant was utilized for subsequent measurement of SOD, GSH, MDA and NO according to earlier reported methods (Adil et al., 2015[1]; Honmore et al., 2015[14]). The remaining portion of the liver supernatant was utilized for subsequent measurement of HDL, LDL, TG and Total cholesterol by using commercially available reagent kits according to the procedure provided by the manufacturer (Accurex Biomedical Pvt. Ltd., Mumbai, India). One liver tissue from each group was processed for histopathological examination.

### Liver tissue histopathology

Liver tissue was fixed in 10 % (v/v) neutral buffered formalin for 24 h for histopathological studies. It was processed for 12 h using isopropyl alcohol, xylene, and paraffin embedded for light microscopic study (Nikon E200, Japan). Paraffin-embedded tissue sections cut at 5 μm thickness were prepared and stained after deparaffination using hematoxylin and eosin stain (H & E) to verify morphological assessment. Photomicrographs were captured at a magnification of 40X.

### Statistical analysis

All statistical analysis was performed using GraphPad Prism 6.0 (GraphPad Software, Inc, La Jolla, CA, USA). Data of body weight and biochemical measurements was analyzed by separate One-way ANOVA followed by Dunnett's test separately for each parameter. A value of p *< *0.05 was considered to be statistically significant.

## Results

### Characterization of ferulic acid by IR,^1^H NMR, ^13^C NMR and LC-MS

**Figure 1:**
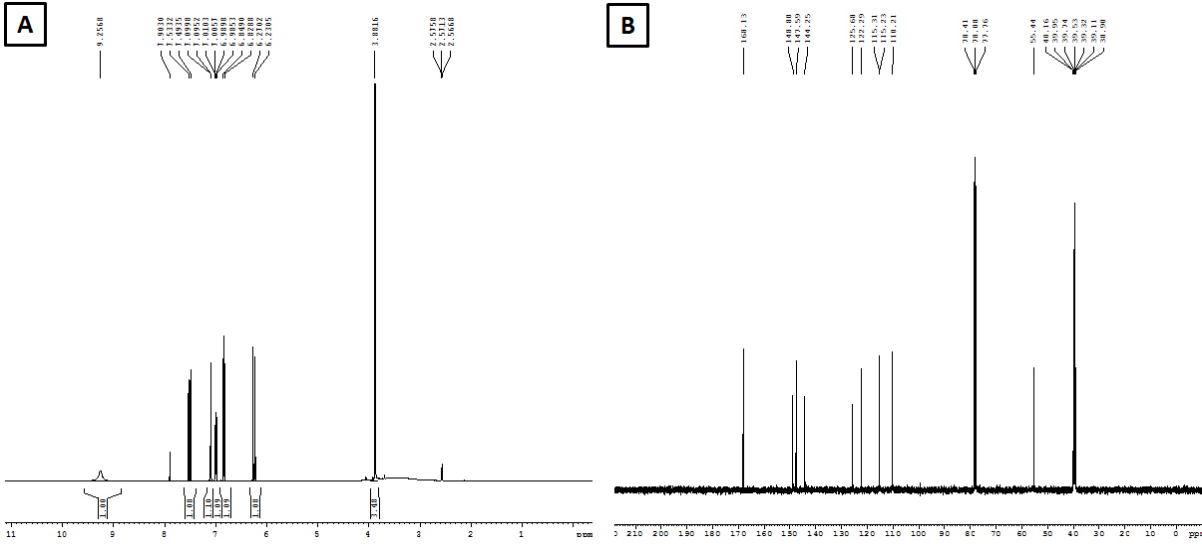
^1^H NMR spectrum of ferulic acid (A) and ^13^C NMR spectrum of ferulic acid (B) were recorded using CDCl_3_ as a solvent on Bruker Advance II 400 NMR. ^1^H NMR spectrum showed the presence of three methyl groups at δ 3.9 s, (3H, J¼5.0, 10.0 Hz), 6.2 d, (1H, J¼7.0, 10.0 Hz) of CH, 6.8 d, (1H, CH of ar. ring), 6.9 d, (1H, CH of ar. ring), 7.1 s, (1H, CH), 7.5 d, (1H, CH) and 9.2 s, (1H, OH) for hydroxyl group, whereas ^13^C NMR spectrum showed 10 signals for 10 carbon signals.

**Figure 2:**
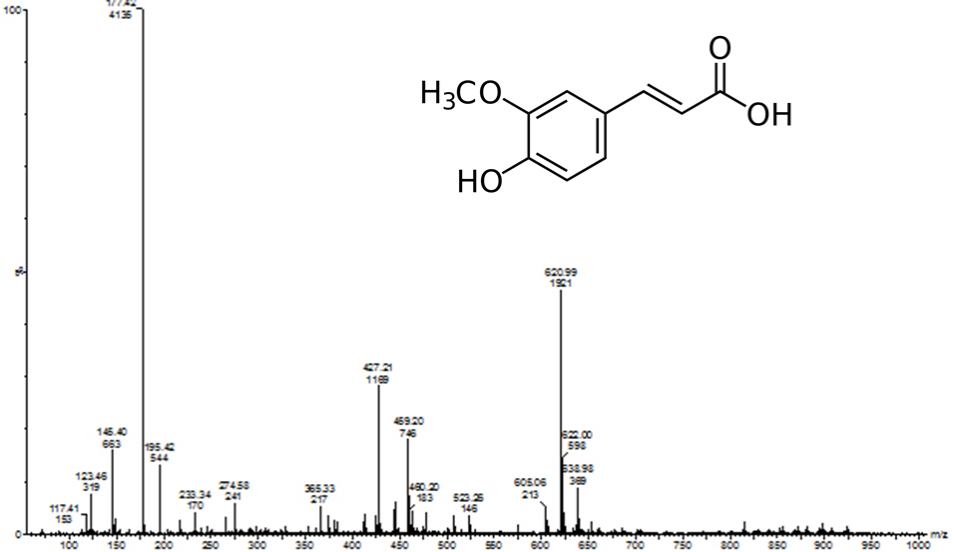
LC-MS spectrum of ferulic acid showed the molecular ion peak M+1 peak at m/z. 195.42 suggesting one of the possible molecular formula as C_10_H_10_O_4_.

The chemical structure of the isolated compound was elucidated by infra-red spectroscopy (IR), proton nuclear magnetic resonance (^1^H NMR), carbon nuclear magnetic resonance (^13^C NMR), and Liquid chromatography-mass spectrometry (LC-MS) experiment. The characterization of isolated compound was carried out by using FT-IR, ^1^H NMR (Figure 1A(Fig. 1)), ^13^C NMR (Figure 1B(Fig. 1)) and LC-MS spectra (Figure 2(Fig. 2)). This characterization results suggested that the isolated compound consists of a single chemical moiety with 93 % purity. The isolated compound obtained was crystalline light yellow color solid (melting point 168 °C to 172 °C). 

In its LC-MS, the molecular ion peak M+1 peak at m/z. 195.42 suggesting one of the possible molecular formula as C_10_H_10_O_4_. Its IR spectrum showed a characteristic broad absorption band at 3438 cm^-1^ for the hydroxyl group, it further showed broad peaks at 2968 cm^-1^ for the carboxylic acid group, 1691 cm^-1^ for the carbonyl group and 1275 cm^-1^ for the C-O group. 

Its ^13^C NMR spectrum showed 10 signals for 10 carbon signals in the following form: (a) nine CH, (b) one CH_3_.

The ^1^H NMR spectrum further showed the presence of three methyl groups at δ 3.9 s, (3H, J¼5.0, 10.0 Hz), 6.2 d, (1H, J¼7.0, 10.0 Hz) of CH, 6.8 d, (1H, CH of ar. ring), 6.9 d, (1H, CH of ar. ring), 7.1 s, (1H, CH), 7.5 d, (1H, CH) and 9.2 s, (1H, OH) for hydroxyl group. From the NMR and LC-MS data, the structure of the compound was confirmed to be ferulic acid (Singh et al., 2013[45]) with purity of 93 %. 

### Effect of ferulic acid on HFD-induced alteration in body weight and liver weight of rats

The body weight and liver weight was significant increased (p *< *0.001) in the HFD control rats as compared to the normal group (Table 1(Tab. 1)). However, increased body weight and liver weight was significantly decreased (p *< *0.001) by FA (20 and 40 mg/kg) treatment as compared to HFD control rats (Table 1(Tab. 1)). Atorvastatin (1.2 mg/kg) treatment also produces a significant decrease (p *< *0.001) in body weight as well as liver weight when compared to HFD control rats. Moreover, body weight and liver weight was more significantly decreased (p *< *0.05) by treatment with FA (40 mg/kg) treatment as compared to atorvastatin (1.2 mg/kg) treatment (Table 1(Tab. 1)).

### Effect of ferulic acid on HFD-induced alteration in serum lipid profile and atherogenic index of rats

Administration of HFD significantly increases (p *< *0.001) the serum triglyceride, cholesterol, LDL-C, VLDL-C, LDL to HDL ratio and atherogenic index in the HFD control rats as compared to normal rats (Table 1(Tab. 1)). Administration of FA (10, 20 and 40 mg/kg) caused a significant and dose-dependent decrease (p *< *0.01, p *< *0.001 and p *< *0.001) in serum triglyceride, cholesterol, LDL-C, LDL to HDL ratio and atherogenic index as compared to HFD control group (Table 1(Tab. 1)). 

There was a significant decrease (p < 0.001) in the serum HDL-C of HFD control group when compared to normal control group (Table 1(Tab. 1)). Treatment with FA (20 and 40 mg/kg) significantly and dose-dependently inhibited (*p* < 0.01 and *p* < 0.001) HFD-induced decreased serum HDL-C as compared to HFD control group (Table 1(Tab. 1)). When compared with HFD control group, atorvastatin (1.2 mg/kg) treatment also produced a significant inhibition (*p* < 0.001) in the HFD induced alteration in serum triglyceride, cholesterol, HDL-C, LDL-C, LDL to HDL ratio and atherogenic index as compared to HFD control group. Moreover, serum LDL-C level and LDL to HDL ratio was more significantly decreased (p *< *0.05) by FA (40 mg/kg) treatment as compared to atorvastatin (1.2 mg/kg) treatment (Table 1(Tab. 1)).

### Effect of ferulic acid on HFD-induced alteration in hepatic lipid profile and atherogenic index of rats

HFD control rats exhibits a significantly increased (p *< *0.001) hepatic triglyceride, cholesterol, LDL-C, VLDL-C, LDL to HDL ratio and atherogenic index whereas HDL-C level in liver was decreased significantly (p *< *0.001) as compared to normal rats (Table 2(Tab. 2)). Administration of FA (20 and 40 mg/kg) resulted in a significant inhibition(p *< *0.05) in HFD induced alterations in hepatic triglyceride, cholesterol, HDL-C, LDL-C, VLDL-C, LDL to HDL ratio and atherogenic index (Table 2(Tab. 2)). Atorvastatin (1.2 mg/kg) treatment also produced a significant inhibition (p *< *0.001) in the HFD-induced alterations in hepatic triglyceride, cholesterol, HDL-C, LDL-C, VLDL-C, LDL to HDL ratio and atherogenic index as compared to HFD control group. Furthermore, FA (40 mg/kg) treatment more significantly decreased (p *< *0.05) the level of hepatic cholesterol as compared to atorvastatin (1.2 mg/kg) treatment (Table 2(Tab. 2)).

### Effect of ferulic acid on HFD-induced alteration in hepatic SOD, GSH, MDA and NO levels of rats

**Figure 3:**
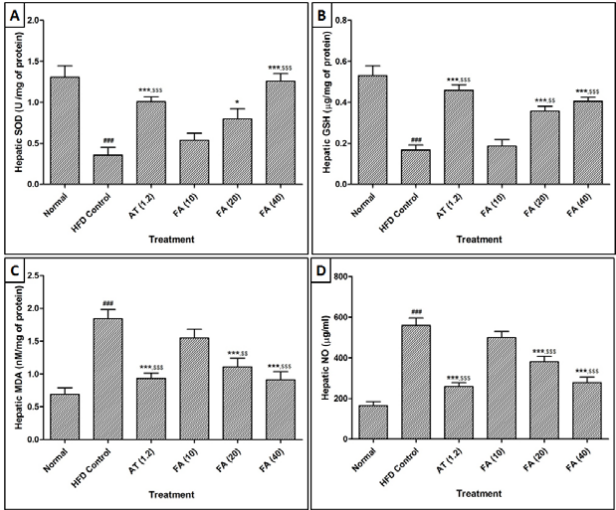
Effect of ferulic acid on HFD-induced alteration in hepatic superoxide dismutase (SOD) (A), Glutathione (GSH) (B), Malondialdehyde (MDA), and (C) Nitric oxide (NO) of rats Data are expressed as Mean ± SEM and analyzed by One-Way ANOVA followed by Dunnett's post-tests. ^###^p *< *0.001 as compared with normal group, *p *< *0.05, ***p *< *0.001 as compared with HFD control group and ^$^p *< *0.05, ^$$^p *< *0.01, ^$$$^p *< *0.001 as compared with ferulic acid treated group. HFD: High Fat Diet, AT (1.2): atorvastatin (1.2 mg/kg, p.o.) treated, FA (10): Ferulic acid (10 mg/kg) treated rats, FA (20): Ferulic acid (20 mg/kg) treated rats and FA (40): Ferulic acid (40 mg/kg) treated rats (n = 6)

There was a significant decrease (p *< *0.001) in the hepatic SOD and GSH level whereas MDA and NO levels in liver were increased significantly (p *< *0.001) in HFD control group as compared to normal group (Figure 3(Fig. 3)). Treatment with FA (20 and 40 mg/kg) significantly increased (p *< *0.001) hepatic SOD and GSH levels as compared to HFD control rats (Figure 3(Fig. 3)). Administration of atorvastatin (1.2 mg/kg) also produced a significant increase (p *< *0.001) in hepatic SOD and GSH levels as compared to HFD control rats. Furthermore, FA (40 mg/kg) treatment more significantly increased (p *< *0.05) the level of hepatic SOD level as compared to atorvastatin (1.2 mg/kg) treatment (Figure 3(Fig. 3)). 

Administration of FA (20 and 40 mg/kg) significantly inhibited (p *< *0.001) HFD-induced increase in hepatic MDA and NO levels as compared to HFD control rats. Treatment with atorvastatin (1.2 mg/kg) also resulted in a significant decrease (p *< *0.001) in hepatic MDA and NO levels when compared with HFD control rats. Moreover, hepatic MDA level was more significantly decreased (p *< *0.05) by FA (40 mg/kg) treatment as compared to atorvastatin (1.2 mg/kg) treatment (Figure 3(Fig. 3)).

### Effect of ferulic acid on HFD-induced alteration in rat liver histology

**Figure 4:**
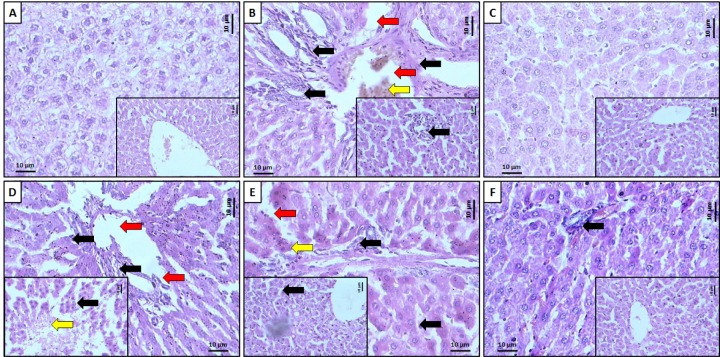
Effect of ferulic acid on HFD-induced alteration in rat liver. Representative photographs of rat liver from each group viz., normal (A), High Fat Diet control (B), Atorvastatin (1.2 mg/kg, p.o.) treated (C), Ferulic acid (10 mg/kg) treated rats (D), Ferulic acid (20 mg/kg) treated rats (E) and Ferulic acid (40 mg/kg) treated rats, (F) necrosis (red arrow), inflammatory infiltration (black arrow) and edema (yellow arrow). H&E staining at 40X and 100X (inset)

Hepatic tissue from normal rats exhibited normal central vein in liver parenchymal cells, without any signs of inflammation and necrosis (Figure 4A(Fig. 4) and Table 3(Tab. 3)). However, it is evident with passive congestion (grade 1) (which may be due to mild dilatation of central veins) and vacuolization (grade 1). The histopathological examination of the liver of HFD control rats reflected a presence of inflammatory cells (black arrow, grade 3) with the presence of vesicular fat (grade 3). Chronic administration of HFD caused hepatocellular injury reflected by the presence of diffuse cytoplasmic vacuolization (grade 3), centrilobular necrosis (red arrow, grade 3), vascular congestion and edema (yellow arrow, grade 2), and nuclear pyknosis (grade 2) of the hepatocytes (Figure 4B(Fig. 4) and Table 3(Tab. 3)). However, atorvastatin (1.2 mg/kg) treatment resulted in mild histopathological changes in liver marked by vesicular fat (grade 1), vacuolization (grade 1), and its devoid of any inflammatory cells infiltration as well as necrosis, congestion, and edema (Figure 4C(Fig. 4) and Table 3(Tab. 3)). In the FA (10 and 20 mg/kg) treated rats, the histology of liver exhibited a moderate degree of vacuolization (grade 2), necrosis (red arrow, grade 2), vesicular fat (grade 2), congestion and edema (yellow arrow, grade 2) around central vein.

Moderate to mild inflammatory cells (black arrow, grade 2) were present in FA (10 and 20 mg/kg) treated rats (Figure 4D(Fig. 4) and Figure 4E(Fig. 4), respectively and Table 3(Tab. 3)). Liver tissue from FA (40 mg/kg) treated rat reflected the presence of inflammatory cells infiltration (black arrow, grade 1), vacuolization (grade 1) and its devoid of any vesicular fat deposition as well as necrosis, congestion and edema (Figure 4F(Fig. 4) and Table 3(Tab. 3)).

## Discussion

Ferulic acid (FA) is a phenolic compound and a major constituent of an array of fruits, vegetables, beverages, and grains. FA serves as a scavenger of hydroxyl and peroxyl radicals that account for its protective function. Previously, the ethanol extract of *P. cineraria* showed potent anti-hyperlipidemic action against high-fat-diet-induced hyperlipidemia in laboratory rat (Jain and Surana, 2016[17]). However, phytoconstituents responsible for its antihyperlipidemic potential is not yet evaluated. It has been reported that FA is insoluble in water at room temperature but it is soluble in hot water, ethyl acetate, ethanol and ethyl ether, and it has been found that ethanol (60 %) is suitable for the successful extraction of FA (Guo et al., 2003[13]). Furthermore, approximately 80 % of the ferulic acid was found in the ethanol extract of bran (Rybka et al., 1993[42]). Ferulic acid isolated by supercritical CO_2_ extraction from ethanol *Angelica sinensis *(Oliv.) Diels extract showed potent antioxidant potential (Sun et al., 2006[48]). Furthermore, for extraction of phenolic compounds from various sources requires 80 % ethanol (Khoddami et al., 2013[28]). Thus, in the present investigation, we have isolated a single compound from the ethanol extract of *P. cineraria*. The physical and chemical methods were utilized to identify and determine the chemical structure of a single isolated compound from the ethanol extract of *P. cineraria*. Based on its physical properties and spectroscopic data (i.e., ^1^H NMR and ^13^C NMR), LC-MS, and IR, the isolated compound was characterized as ferulic acid, (4-hydroxy-3-metoxybenzene acrylic acid), a phenolic compound with a molecular mass of 194.42 daltons, and a molecular formula of C_10_H_10_O_4 _(Figure 2(Fig. 2)). The obtained spectral data were compared with the reported spectral data by other research groups (Islam et al., 2008[16]; Singh et al., 2013[45]). On the basis of chemical characterization of FA, the isolated compound from the ethanol extract of *P. cineraria,* was confirmed as a single component in pure form. Kumar and Pruthi (2014[32]) found the highest known concentration of ferulic acid glucoside in flax seed (4.1 ± 0.2 g/kg), however, in present investigation 10.8 g/kg of ferulic acid (93 %) was isolated from the ethanol extract of *P. cineraria*. Furthermore, we also evaluated the hypolipi-demic potential of FA using the validated animal model of High Fat Diet (HFD) induced hyperlipidemia in Sprague-Dawley rats.

HMG-CoA reductase is an important enzyme in cholesterol biosynthesis and diet enriched with saturated fatty acids increases the activity of HMG-CoA reductase via increased availability of acetyl-CoA. Moreover, high amount of cholesterol and saturated fatty acids associated with a downregulation of LDL receptors results in increased serum LDL-C levels (Thirumalai et al., 2014[50]). In HFD induced hyperlipidemia decreased in the activity of Lecithin Cholesterol O-acyltransferase (LCAT) enzyme caused an alteration in the transesterification of cholesterol which in turn modulated the HDL-C activity (Ji et al., 2011[19]). With increased availability of free fatty acids, the triglyceride levels were significantly increased in HFD control group, which leads to decreased hepatic release of lipoprotein and increased esterification of free fatty acids (Thirumalai et al., 2014[50]). VLDL particles are rich triglycerides and smaller than the chylomicrons. VLDL is the primary carrier of triglycerides. In the present investigation, there was a significant increase in the levels of TG, LDL-C and cholesterol level along with decreased in HDL-C level in serum as well as in liver. Administration of FA significantly inhibited this HFD-induced alteration in serum and liver lipid profile. In the present study, serum VLDL levels were notably raised in HFD control group, whereas FA treatment significantly decreased the level of VLDL. Finding of our investigation is in line with the results of previous studies which showed that administration of reduced plasma triglyceride, free fatty acid, and total cholesterol in diabetic rats (Jin Son et al., 2010[20]; Sri Balasubashini et al., 2003[46]). Furthermore, FA has an ability to inhibit cholesterol synthesis via competitive inhibition of HMG-CoA reductase (Kim et al., 2003[30]). Thus, this antihyperlipidemic potential of FA may be due to its ability to inhibit HMG-CoA reductase activity.

In the pathogenesis of HFD-induced hyperlipidemia, increased cellular reactive oxygen species (ROS) played a vital role via increased oxidative stress thus decreasing antioxidant capacity. SOD and GSH are endogenous antioxidant enzymes which play an essential role in detoxification of toxic oxygen radicals (Kandhare et al., 2013[22]). SOD plays an important role in eliminating the superoxide radicals (Kandhare et al., 2012[25]). GSH is a non-enzymatic biological antioxidant which plays a significant role in quenching of free radical species (such as hydrogen peroxide, superoxide and alkoxy radicals) act as a free radical scavenger (Kandhare et al., 2014[26]). There was a subsequent increase in lipid peroxidation (MDA) with increased production of free radicals which was evident from the reduction of antioxidant status of HFD control rats (Aydin, 2015[2]; Kandhare et al., 2015[21]). Elevated MDA levels along with decreased levels of GSH and SOD mirrors with the toxic effects of ROS produced by HFD, which is consistent with previously reported observations where HFD-administration caused a marked reduction in tissue antioxidant level (Aydin, 2015[2]). Whereas, administration of FA significantly inhibited HFD induced alter oxidative stress which may be due to its antioxidant potential. Findings of the present investigation are in line with results of the previous reports where treatment with FA improved liver antioxidant status (Balasubashini et al., 2004[3]). 

It has been reported that elevated NO production played a vital role in the induction of various diseases including hyperlipidemia (Aydin, 2015[2]). Tissue injury occurred when NO reacts with superoxide and forms peroxynitrites, and it is necessary to inhibit this NO production to ameliorate tissue injury (Kandhare et al., 2013[23], 2012[24]). In the present investigation, HFD caused significant induction of liver damage via increase in the production of NO. Administration of FA resulted in significant amelioration in HFD-induced elevated NO production. It has been reported that FA modulated the nitric oxide (NO) bioavailability and decreased iNO synthesis (Suzuki et al., 2007[49]). Our result provides credence to the finding of the previous researcher that FA caused inhibition of NO production.

It has been reported that elevated oxidative stress contributes to the development of atherosclerosis-linked metabolic syndrome (Choi et al., 2010[10]). Moreover, high amounts of serum triglycerides are linked to atherosclerosis with increased risk of heart disease and stroke (Ong et al., 2009[40]). HFD-induced hyperlipidemia is associated with altered antioxidant defense mechanisms. Hypercholesterolemia leads to atherosclerosis and inhibition in lipid peroxidation inhibits this atherosclerosis (Yokozawa et al., 2003[52]). In the present investigation, rats administered with HFD exhibited an elevated level of oxidative stress along with LDL-C and triglycerides which in turn elevated atherosclerotic index. However, administration of FA produced a significant inhibition in atherosclerotic index via its antioxidative potential. Moreover, findings of previous research showed that phenolic compounds are effective in preventing the formation and progression of atherosclerosis which is mainly due to its antioxidant and hypocholesterolemic potential (Carew et al., 1987[8]). FA is a natural polyphenolic compound, possesses both antioxidant and hypocholesterolemic activity and which might inhibit atherosclerosis progression.

Atorvastatin is an effective agent with significant lipid-lowering potential widely used in the treatment of hyperlipidemia, atherosclerosis or cardiovascular complications (such as coronary heart disease) (Stancu and Sima, 2001[47]). It has an ability to inhibit HMG-CoA reductase enzyme, an enzyme which generates mevalonate via conversion from HMG-CoA. This inhibition of HMG-CoA reductase enzyme serves as a rate-limiting step in the biosynthesis of cholesterol. Thus, inhibition of HMG-CoA reductase, in turn, causes a reduction in cholesterol, LDL-cholesterol and total cholesterol levels (Davignon et al., 1992[11]). However, they have the ability to reduce triglyceride concentrations which depends on the baseline triglyceride levels. However, in the present investigation, FA exhibited a significant reduction in total cholesterol and triglyceride in serum and also in hepatic tissue.

In conclusion, the chemical structure of the isolated compound was determined by ^1^H NMR, ^13^C NMR and LC-MS experiment which revealed that only a single compound was isolated from an ethanol extract of *P. cineraria* and characterized as FA. Finally, the pure isolated FA was screened by using HFD-induced rat model for its hypolipidemic action using serum and liver lipid parameters. FA (20 and 40 mg/kg) resulted in a significant reduction in TC, TG, LDL, VLDL-C, and a significant increase in HDL-C in serum as well as hepatic tissue. It also resulted in a significant amelioration in hepatic oxido-nitrosative stress induced by HFD. A recent study also showed that FA alleviates metabolic syndrome induced by high-carbohydrate diet in a rat model (Senaphan et al., 2015[44]). Thus, it is concluded that ethanol extract of *P. cineraria* possessed hypolipidemic activity mainly due to FA via inhibition of elevated oxido-nitrosative stress. These results with pure isolated FA and previous results with ethanol extract of *P. cineraria* confirm that *P. cineraria* is beneficial in preventing hyperlipidemia in laboratory animals. However, further study is in progress for the elucidation of actual mechanism of action of FA isolated from *P. cineraria* at the molecular level.

## Declaration of interest

The authors report no conflicts of interest.

## Figures and Tables

**Table 1:**
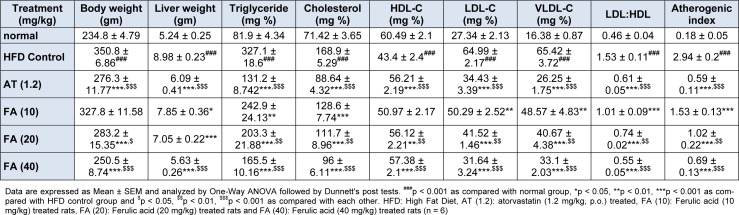
Effect of ferulic acid on HFD-induced alteration in body weight, liver weight, serum lipid profile and atherogenic index of rats

**Table 2:**
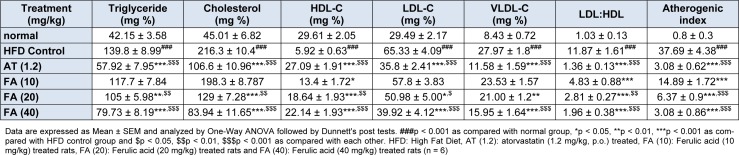
Effect of ferulic acid on HFD-induced alteration in hepatic serum lipid profile and atherogenic index of rats

**Table 3:**
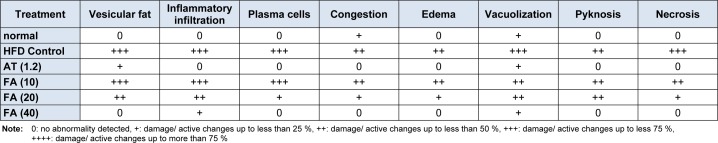
Effect of ferulic acid on HFD-induced alteration in rat liver
